# Voices lost: where is the person in evaluating a palliative approach to care?

**DOI:** 10.1177/26323524231193041

**Published:** 2023-08-28

**Authors:** Richard Sawatzky, Pat Porterfield, Erin Donald, Carolyn Tayler, Kelli Stajduhar, Sally Thorne

**Affiliations:** School of Nursing, Trinity Western University, 22500 University Drive, Langley, BC, V2Y 1Y1, Canada; Centre for Advancing Health Outcomes Sciences, Vancouver, BC, Canada; Independent; School of Nursing & Institute on Aging and Lifelong Health, University of Victoria, Victoria, BC, Canada; British Columbia Centre for Palliative Care, Vancouver, BC, Canada; School of Nursing & Institute on Aging and Lifelong Health, University of Victoria, Victoria, BC, Canada; School of Nursing, University of British Columbia, Vancouver, BC, Canada

**Keywords:** narrative review, palliative approach, person-centredness, outcomes evaluation

## Abstract

Person-centredness is a cornerstone to a palliative approach to care. However, there is a risk that a person-centred perspective is lost in how a palliative approach is evaluated. We explored the extent to which evaluations of a palliative approach are consistent with its person-centred ethical stance. Using a narrative review approach, we critically reflected on how the experiences, priorities and concerns of patients and family are represented, or not represented, in evaluations of a palliative approach. We were guided by the following questions: (1) What types of outcomes and indicators are commonly used to evaluate a palliative approach? (2) Whose perspectives are represented in current evaluations of a palliative approach? And (3) What are the foci of evaluation in this body of research? We observed that the evaluations of a palliative approach are commonly based on indicators of its implementation and predominantly reflect the perspectives of healthcare providers and healthcare systems, rather than patients or family. Although evaluations focused on healthcare providers and systems are important for integrating a palliative approach, there is concern that the essence of person-centredness is lost when the perspectives of patients and families about their healthcare needs, outcomes and experiences are not consistently measured as the ultimate goal of care. There is a need for more emphasis on evaluation practices that value person-centred outcomes, in addition to outcomes oriented to the needs of healthcare providers and systems.

## Introduction

The World Health Organization defines palliative care as an approach that improves the quality of life of patients and their families facing the problems associated with life-threatening illness. This is accomplished through the prevention and relief of suffering by means of early identification and comprehensive assessment and treatment of pain and other problems – physical, psychosocial and spiritual. The WHO further states: ‘Palliative care is a crucial part of integrated, people-centred health services’.^1^

Although palliative care was defined as an approach for all those facing life-limiting illness, the initial populations receiving palliative care were predominantly persons living with malignant disease. Driven by the need to develop accessible high-quality end-of-life care for all who stand to benefit from it, the idea of a more widespread palliative approach to care has been constructed.^2,3^ Although a palliative approach applies along the entire trajectory of life-limiting illness, from experiencing early symptoms to dying and grief, it is distinct from specialized palliative care. A palliative approach is conceptualized as an upstream orientation towards the needs of people who have life-limiting conditions and their families: it adapts the principles, knowledge and skills of palliative care to all life-limiting illnesses and conditions, and it integrates them into non-specialist palliative care settings.^4^

A palliative approach often melds chronic disease management with a person-centred (also referred to as patient-centred) approach focused on quality of life for the person and not just the medical care of the disease.^5^ From the person’s perspective, this person-centred approach involves being listened to, being informed and having a say in one’s care.^3,6^ From the perspective of palliative care providers, a person-centred approach has been described as ‘respectful of, and responsive to, the needs, preferences and values of the person receiving care, their family, and other caregivers’.^7^ Communication and the relationship between the person and the healthcare provider, characterized as a partnership, are central to person-centred care.^6,8,9^ Within the healthcare system, person-centred care can be viewed as an ethical stance focused on caring for the individual as a person and promoting ‘just institutions’ as reflected in the services, organization and evaluation of care.^10,11^

Although much work has focused on introducing and integrating a palliative approach into healthcare systems,^12^ there has been a concurrent and increasing need to evaluate its implementation and impact. However, competing agendas influence the need to evaluate a palliative approach at micro-, meso- and macro-levels of the health system.^13^ This has led to the development of multiple standardized indicators, metrics and tools for evaluation. At the micro-level, evaluations are predominantly concerned with quality of life, desires and needs of patients and family members as the basis for shared decision-making and care planning. Meso-level considerations emphasize organizational priorities focused on monitoring, evaluating and improving the implementation of a palliative approach in care delivery. At the macro-level, the emphasis is on evaluation of population health considerations including regulation, adherence to policies and measurement of cost-efficiency. As a result of these competing agendas, there is a risk for the person-centred perspective to be lost in evaluations of a palliative approach.

Given that a palliative approach is to be person-centred, yet integrated within healthcare systems, it is important that evaluations reflect the diverse perspectives of *all* people receiving care as well as indicators of care delivery and healthcare system performance. Our research was motivated by the desire to identify to what extent person-centredness is actually represented in how a palliative approach is being evaluated and to critically reflect on these potentially competing perspectives.

To explore the extent to which evaluations of a palliative approach are consistent with its person-centred ethical stance, we conducted a review of evaluation practices reported in empirical literature. We used the following questions to guide our analysis:

(1) What types of outcomes and indicators are being commonly used to evaluate a palliative approach?
(2) Whose perspectives are represented in current evaluations of a palliative approach?
(3) What are the foci of evaluation in this body of research?

The answers to these questions led to our critical reflection on how the experiences, priorities and concerns of patients and family are represented, or not represented, in the outcomes and indicators used to evaluate a palliative approach.

## Narrative review approach

Considering our objective towards critical reflection rather than systematic documentation, we employed a narrative review approach focused on constructing a ‘scholarly summary along with interpretation and critique’.^14^ We identified relevant manuscripts from our original review on a palliative approach (which only included articles from prior to 2012) and conducted an updated search (2012–2019) of the same library databases using the terms ‘palliative approach’ or ‘palliative care approach’. The resulting 528 articles were screened to identify those that discuss outcomes or indicators used to evaluate a palliative approach. Consistent with our conceptualization of a palliative approach, we did not include articles that focused only on: (a) the final days or hours of life, (b) the treatment of a particular symptom or (c) specialized palliative care. We included 88 studies focused on adult populations, which were written in English, and those that reported on a primary study (including protocols; see Appendix for list of included articles). Guided by the above three research questions, we captured information about (a) the outcomes or indicators used to evaluate a palliative approach, (b) the data sources for evaluation (i.e. administrative data; chart review data; data from healthcare providers; and data from patients, family, or friends) and (c) the intervention or initiative being evaluated. We employed a constant comparative analytical approach to clarify, expand on, refine and organize the initial conceptual groupings we observed, followed by a critical reflective process to ask interpretive questions of what we saw in that body of literature.

### What outcomes and indicators are used to evaluate a palliative approach?

**Figure 1.**
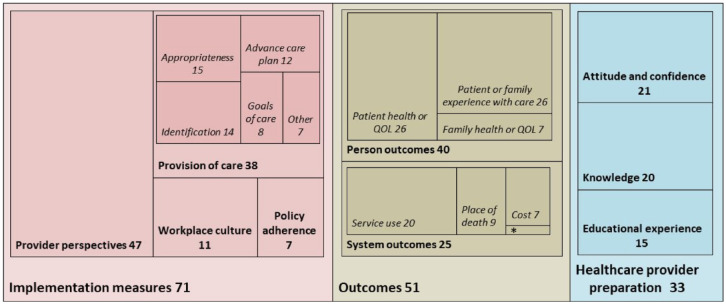
Relative frequencies of articles that refer to preparation, implementation or outcome measures. Box sizes are proportional to the number of articles within each level of the hierarchy chart. *Job satisfaction (*n* = 1).

Overall, the 88 articles revealed 3 overarching categories of different indicators and outcomes: (a) healthcare provider outcomes regarding interventions to prepare them for delivering a palliative approach, (b) indicators (including process indicators) of a palliative approach implementation and (c) outcomes reflecting the results of implementation (see Figure 1). Most articles (55) addressed indicators or outcomes in more than one of these categories, including 12 articles that included all three categories.

A palliative approach was most commonly evaluated based on indicators of its implementation (*n* = 71), the largest category being the healthcare providers’ experiences of care (*n* = 47). To illustrate, in one study district nurses were interviewed about ‘how they perceived their role in supporting cancer patients with palliative care needs at home’ (Griffiths *et al.*, p. 157).^15^ Another study used questionnaires in residential care to measure ‘nurse assistants experiences of care provision and caring climate’ (Beck *et al*., p. 830),^16^ and yet another study used focus groups, interviews and a survey of medical and nursing staff to evaluate transitions to palliative care (Gott *et al*).^17^ Further, 38 articles included implementation outcomes focused on aspects of care provision, such as: advance care planning, identification of people who would benefit from a palliative approach, goals of care conversations and symptom assessment interventions. Several articles (11) evaluated workplace culture (e.g. relationships and communication between care providers) as an indicator of whether a palliative approach was being successfully implemented. Relatively fewer articles (7) focused on adherence to healthcare system policies or guidelines for implementing a palliative approach. Additionally, 33 articles focused on preparing healthcare providers to deliver a palliative approach, including evaluations of healthcare provider attitudes or confidence (21), knowledge (20) and the provider perspectives on their experiences with educational programmes (15).

Outcomes resulting from a palliative approach were evaluated in just over half of the articles (51). This includes 40 articles that evaluated patient- and family-centred health and quality of life outcomes, and 25 that evaluated healthcare system outcomes. Patient health (in particular symptoms and functioning) and quality of life were evaluated as outcomes of a palliative approach in only 26 of the articles (e.g. using standardized quality of life assessment tools or patient-reported outcome measures), and 7 articles evaluated health and quality of life of family members. Experience of care (as perceived by patients or family) was evaluated in 26 of the articles. For example, one study protocol involved use of the Schedule for the Evaluation of Individual Quality of Life, the Palliative care outcome Scale-Symptoms-MS, and the Core Palliative care Outcomes Scale to evaluate impact of a home-based palliative approach on overall quality of life, symptoms, and ‘emotional, psychological and spiritual needs, and provision of information and support’.^18^ In addition, system-level indicators focusing on cost, place of death and service use were evaluated in 7, 9 and 20 of the articles, respectively.

#### Critical reflection

Overall, the above observations indicate an emphasis on indicators regarding the *process* of delivering a palliative approach and less emphasis on *outcome* measures for evaluating the results of a palliative approach. In addition, delivery is more often evaluated from the healthcare providers’ point of view; many articles focus on healthcare providers’ care experiences or elements of care provision, whereas patient- and family-centred outcomes are less prominently measured. Given that person-centredness is a core value embedded within a palliative approach to care, important questions arise, such as: Why are patient- and family-centred outcomes not more routinely evaluated? How are patients’ and families’ voices accounted for in evaluations of a palliative approach? Of the articles that did describe outcomes, what are the motivations for measuring system outcomes, including service use and cost? To what extent are we using these system outcomes to advance a person-centred perspective? Although the WHO sees palliative care as integral to a people-centred integrated healthcare system, these reflections highlight the need for more attention to measuring outcomes and indicators from a person-centred point of view.

### Whose perspectives are represented in evaluations of a palliative approach?

**Figure 2.**
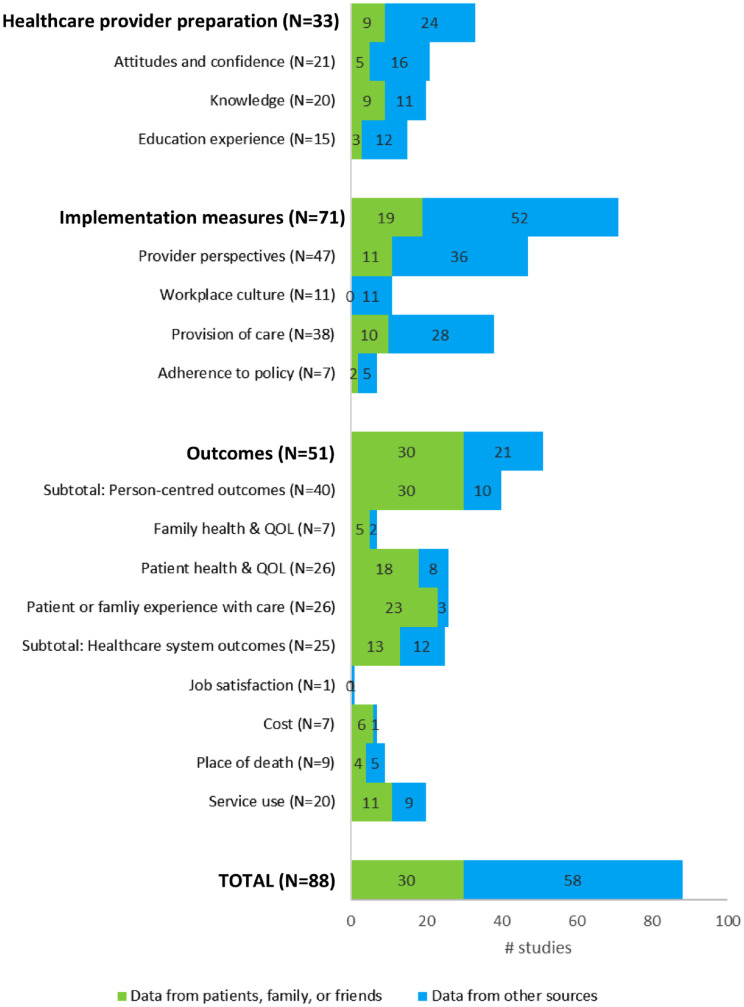
Data sources by outcomes or indicators.

Regarding the data sources of the 88 studies, 12 studies utilized administrative health data, 24 utilized chart review data, and 64 utilized data obtained from healthcare providers. Conversely, only 30 studies included data that were reported directly by patients or family, whereas the remaining 58 studies included data only from sources (administrative, chart review, healthcare providers) for each of the palliative approach outcomes or indicators (see Figure 2). Although there were 40 studies that reported on patient- and family-centred outcomes, only 30 of these included data directly reported by patients or families. The remaining 10 studies relied upon administrative datasets (1 study), chart reviews (3 studies) or healthcare providers (6 studies) for evaluating patient- and family-centred outcomes. When further considering the perspectives that are brought to the evaluation, another important question is the extent to which patients or families were engaged in the design and implementation of evaluation procedures. Indeed, only 11 studies reported any evidence of patient- or family-engagement, for example, being part of a study advisory committee, construction of measurement tools and approaches, or broader community participation in the design and implementation of the study.

#### Critical reflection

The findings from this narrative literature review show that a palliative approach has been predominantly evaluated using data from providers and healthcare systems. Although measures of person-centredness can be derived from various data sources, it is nonetheless important to ensure that, where possible, measures of how patients and families are doing (i.e. their outcomes) and how they are experiencing the care they received are obtained directly from them.^19,20^ These results lead to important questions about the representation of patients and families in evaluations of a palliative approach. Why are patients and families not more routinely included in studies focused on their healthcare experiences and outcomes? Whose voices are not well-represented because of these evaluative practices? Clearly, there is a critical need to develop strategies and means to ensure inclusion of diverse patients and family perspectives.

### What was being evaluated using which outcomes?

**Figure 3.**
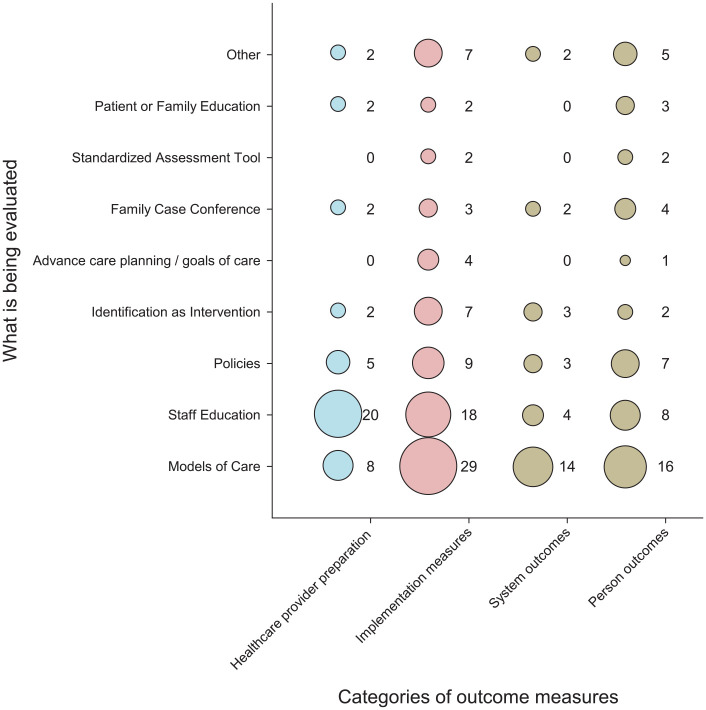
Relationship between what is being evaluated and outcome measures used (# articles).Colors match the 3 categories of different indicators and outcomes as per Figure 1.

Studies evaluating models of care were most frequent (*n* = 34), followed by staff education or community of practice initiatives *(n* = 24), and policies related to the implementation of a palliative approach (*n* = 12). Relatively few studies focused on evaluating specific palliative approach interventions (see Figure 3). For example, only seven studies evaluated identification as a specific intervention, and only four studies focused family case conferences. However, these and other specific interventions were often contained within the overarching category ‘model of care’. Studies were classified as focusing on a ‘model of care’ when the care delivery system described both clinical practice initiatives and integration of a palliative approach within the existing healthcare system. The most prevalent model of care was the UK Gold Standards Framework (GSF),^21^ with eight studies focusing on GSF in Primary Care and three on GSF in Nursing Homes. The remaining 23 studies described models for care delivery organized around a particular condition (e.g. chronic obstructive pulmonary disease or multiple sclerosis), or a particular practice setting, such as rural nursing or long-term care.

Understandably, most studies focusing on staff education initiatives included evaluations based on healthcare provider outcomes (*n* = 20), such as their attitudes, knowledge and confidence, in addition to indicators of palliative approach implementation (*n* = 18), healthcare system outcomes (*n* = 4) and patient- and family-centred outcomes (*n* = 8). By comparison, most of the 34 studies focusing on models of care included evaluations based on indicators of palliative care implementation (*n* = 29), in addition to patient- and family-centred outcomes (*n* = 16), healthcare system outcomes (*n* = 14) and, to some extent, HCP provider outcomes (*n* = 8; see Figure 3).

#### Critical reflection

A palliative approach has evolved to address real shortcomings in healthcare, which include attending to the goals, wishes and needs of people living with life-limiting illness and their participation in decisions about their care. Evaluative practices predominantly reflect an assumption that addressing these shortcomings requires educational programmes and new models of care with evaluations focused mostly on implementation. But why are these models predominantly evaluated in the absence of person-centred outcomes, including feedback from patients and families? Is the central nature of person-centredness being lost in how a palliative approach is being evaluated? Does an emphasis on models reflect a preoccupation with healthcare systems and less so a person-centred ethical stance involving relational practice? Models of care and educational strategies are important vehicles for facilitating changes in how healthcare is provided. However, there is an equally important need for measures and indicators that demonstrate the embeddedness of person-centredness in the provision of a palliative approach.

## Discussion

The findings from our review and critical reflections suggest that evaluations of a palliative approach are heavily influenced by an orientation towards healthcare systems with emphasis on staff education and models of care. Although evaluations focused on healthcare systems are important for integrating a palliative approach, there is concern that the essence of person-centredness is lost when the perspectives of patients and families about their healthcare needs, outcomes and experiences are not measured as the ultimate goal of care.

Person-centredness is a broadly used concept that is widely regarded as a cornerstone of health care.^6,20^ While not exclusive to a palliative approach to care, it is essential to it.^4^ Indeed, improved quality of life of patients and their family, as well as improved experiences with their care, are the overarching goals of both person centredness and a palliative approach.^6,10^ However, measuring these goals must be grounded in an understanding of person-centredness as both a philosophy and a complex multi-layered strategy that permeates healthcare systems.^20^

For the purposes of deepening our understanding of person-centredness within a palliative approach, the following three major themes identified in a systematic overview of reviews are very applicable: (1) the attributes of centredness (what it is), (2) how centredness is translated into practice (how it is done) and (3) evaluation of the effects (possible ways of measuring the effects of centredness).^11^ The review further identified three key attributes of centredness. Firstly, ‘being unique’ recognizes that although persons share basic needs and rights, they have unique personalities and responses to illness. Secondly, ‘being heard’ relates to the opportunities to share their unique stories, needs and preferences in a respectful way. And thirdly, ‘shared responsibility’ involves the person’s participation in choices, decisions and care. These attributes are represented to varying degrees in measures of patient and family experience and satisfaction with care. However, as shown in our results, these are not the most common outcomes and not always gathered directly from patients and families.

The second theme of centredness, translation into practice, draws attention to the interactions and relationships between the person, the family and the healthcare providers.^11^ Although care processes, including communication and shared decision-making, are central to a palliative approach, challenges arise when these are reduced to standardized guidelines and checklists that are not individualized. For example, implementation of a palliative approach has focused on criteria for identifying people who need a palliative approach and the use of practice guidelines for discussions such as goals of care. However, person-centredness requires flexible approaches that tailor the application of such standardized guidelines and tools to the person’s preferences and capabilities to engage in care planning and decision-making. Process evaluation of a palliative approach therefore requires evidence from patients and families of the extent to which such tailoring is being addressed in educational interventions and the implementation of guidelines and tools.^6^

In addition to evaluations focused on palliative approach implementation, evaluations of centredness are needed to determine whether the implementation is having the desired impacts. According to Feldthusen *et al.*,^11^ such evaluation should focus on health-related effects (health status); person self-evaluations (e.g. quality of life, functioning, symptoms, feeling validated and understood); family participation and experiences; and organizational effects (e.g. access to care, quality and safety and readmission rates). While the importance of improving our measurements of quality of life and healthcare experiences of patients and families is widely acknowledged, person centredness further challenges us to shift the prevailing focus on measuring what matters most on average to findings ways of conducting evaluations that capture uniqueness – diverse healthcare needs, outcomes and experiences.

Collectively, the themes and attributes of centredness point to the need to integrate commonalities and individual differences in our evaluations of a palliative approach. Whereas the predominant emphasis in healthcare system evaluation practices has been on nomothetic measurements that focus on population averages, idiographic assessments and measurements of individual differences are needed to understand how, whether and for whom a palliative approach is achieving desired person-centred outcomes.^10,22^ Guided by the centredness attributes of ‘being unique’, ‘being heard’ and ‘shared responsibility’, we need to shift our focus on measuring what *typical* patients might want on average (nomothetic point of view) towards revealing diverse perspectives of *particular* patients (idiographic point of view). This motivation becomes increasingly relevant and important as our systems are being challenged to attend to matters of equity in health care – extending our offerings of the typical to meet the needs of the minority, marginalized and far too often underserved among our patient populations.^20^

We believe that health system evaluation practices privilege healthcare provider and system perspectives and indirect indicators rather than tackling the complex and inherently messy question of whether patients and families experience care as individualized, person-centred and relevant to their unique and diverse needs. This may have led to an unintended consequence regarding the generation of a body of knowledge that seems to further support conventional evaluation practices rather than posing the fundamental question upon which a palliative approach is grounded: How will we know if the care we provided is the best possible care for all people who need a palliative approach? How do we ensure that our evaluations lead to interpretations, actions and decisions that are aligned with a person-centred orientation? How do we prevent the unintended consequences of not representing the needs, experiences and outcomes of marginalized and underserved populations? Given that healthcare professionals are collectively committed to person-centred care, we need to construct alternative (or parallel) ways to ask these profoundly complex questions.

## Conclusion

System-oriented evaluation metrics delude us into thinking we have neatly packaged and convenient answers. While there may be many good reasons to continue to collect them, we must not forget the larger purpose for which we are doing this work—the crucial objectives of health care. There may never be a neatly packaged answer to the question of how we would know if we were providing the right care for this patient and family in their season of need. But that does not mean we should ever stop trying to ask it.

## Supplemental Material

sj-docx-1-pcr-10.1177_26323524231193041 – Supplemental material for Voices lost: where is the person in evaluating a palliative approach to care? Click here for additional data file. Supplemental material, sj-docx-1-pcr-10.1177_26323524231193041 for Voices lost: where is the person in evaluating a palliative approach to care? by Richard Sawatzky, Pat Porterfield, Erin Donald, Carolyn Tayler, Kelli Stajduhar and Sally Thorne in Palliative Care and Social Practice

## Declarations

**Ethics approval and consent to participate:** Not applicable.

**Consent for publication:** Not applicable.

**Availability of data and materials:** All data contributing to this review are available upon request to the corresponding author.
