# Supplementary material for: Voices lost: where is the person in evaluating a palliative approach to care?
Source: Palliat Care Soc Pract. 2023 Aug 28;17:26323524231193041. doi: 10.1177/26323524231193041 (PMC10467210; doi:10.1177/26323524231193041)
Supplement: sj-docx-1-pcr-10.1177_26323524231193041 – Supplemental material for Voices lost: where is the person in evaluating a palliative approach to care? [file sj-docx-1-pcr-10.1177_26323524231193041.docx]

**Appendix: Reference list of included sources**

1. Agar M, Beattie E, Luckett T, et al. Pragmatic cluster randomised controlled trial of facilitated family case conferencing compared with usual care for improving end of life care and outcomes in nursing home residents with advanced dementia and their families: The IDEAL study protocol. *Bmc Palliat Care* 2015; 14. DOI: 10.1186/s12904-015-0061-8.
2. Agar M, Luckett T, Luscombe G, et al. Effects of facilitated family case conferencing for advanced dementia: A cluster randomised clinical trial. *PLoS One* 2017; 12: e0181020. 2017/08/09. DOI: 10.1371/journal.pone.0181020.
3. Andrews S, McInerney F and Robinson A. Realizing a palliative approach in dementia care: Strategies to facilitate aged care staff engagement in evidence-based practice. *International Psychogeriatrics* 2009; 21: S64-S68. 2009/03/18. DOI: 10.1017/s1041610209008679.
4. Arcand M, Monette J, Monette M, et al. Educating nursing home staff about the progression of dementia and the comfort care option: Impact on family satisfaction with end-of-life care. *Journal of the American Medical Directors Association* 2009; 10: 50-55. DOI: 10.1016/j.jamda.2008.07.008.
5. Badger F, Clifford C, Hewison A, et al. An evaluation of the implementation of a programme to improve end-of-life care in nursing homes. *Palliative Medicine* 2009; 23: 502-511. DOI: 10.1177/0269216309105893.
6. Beck I, Jakobsson U and Edberg AK. Applying a palliative care approach in residential care: Effects on nurse assistants' experiences of care provision and caring climate. *Scandinavian Journal of Caring Sciences* 2014; 28: 830-841. DOI: 10.1111/scs.12117.
7. Beck I, Jakobsson U and Edberg AK. Applying a palliative care approach in residential care: effects on nurse assistants' work situation. *Palliat Support Care* 2015; 13: 543-553. 2013/10/22. DOI: 10.1017/s1478951513000783.
8. Beck I, Tornquist A and Edberg AK. Nurse assistants' experience of an intervention focused on a palliative care approach for older people in residential care. *Int J Older People Nurs* 2014; 9: 140-150. DOI: 10.1111/j.1748-3743.2012.00343.x.
9. Burgess T, Braunack-Mayer A, Crawford GB, et al. Australian health policy and end of life care for people with chronic disease: An analysis. *Health Policy* 2014; 115: 60-67. DOI: 10.1016/j.healthpol.2013.08.001.
10. Buxton KL, Stone RA, Buckingham RJ, et al. Current and planned palliative care service provision for chronic obstructive pulmonary disease patients in 239 UK hospital units: Comparison with the gold standards framework. *Palliative Medicine* 2010; 24: 480-485. DOI: 10.1177/0269216310363650.
11. Casotto V, Rolfini M, Ferroni E, et al. End-of-life place of care, health care settings, and health care transitions among cancer patients: Impact of an integrated cancer palliative care plan. *Journal of Pain & Symptom Management* 2017; 54: 167-175. DOI: 10.1016/j.jpainsymman.2017.04.004.
12. Dale J, Petrova M, Munday D, et al. A national facilitation project to improve primary palliative care: Impact of the Gold Standards Framework on process and self-ratings of quality. *Quality & Safety in Health Care* 2009; 18: 174-180. DOI: 10.1136/qshc.2007.024836.
13. Davison SN. End-of-life care preferences and needs: Perceptions of patients with chronic kidney disease. *Clinical Journal of the American Society of Nephrology* 2010; 5: 195-204. DOI: 10.2215/CJN.05960809.
14. den Herder-van der Eerden M, van Wijngaarden J, Payne S, et al. Integrated palliative care is about professional networking rather than standardisation of care: A qualitative study with healthcare professionals in 19 integrated palliative care initiatives in five European countries. *Palliative Medicine* 2018; 32: 1091-1102. DOI: 10.1177/0269216318758194.
15. Durepos P, Kaasalainen S, Sussman T, et al. Family care conferences in long-term care: Exploring content and processes in end-of-life communication. *Palliat Support Care* 2018; 16: 590-601. OI: 10.1017/s1478951517000773.
16. Elkington HM, White PT, Higgs R, et al. A palliative care approach in severe COPD: GPs' views of discussing prognosis. *Thorax* 2000; 55: A7-A7.
17. Ersek M, Grant MM and Kraybill BM. Enhancing end-of-life care in nursing homes: Palliative Care Educational Resource Team (PERT) program. *Journal of Palliative Medicine* 2005; 8: 556-566. DOI: 10.1089/jpm.2005.8.556.
18. Ersek M, Kraybill BM and Hansen NR. Evaluation of a Train-the Trainer program to enhance hospice and palliative care in nursing homes. *Journal of Hospice and Palliative Nursing* 2006; 8: 42-49.
19. Finucane AM, Stevenson B, Moyes R, et al. Improving end-of-life care in nursing homes: Implementation and evaluation of an intervention to sustain quality of care. *Palliative Medicine* 2013; 27: 772-778. DOI: 10.1177/0269216313480549.
20. Frey R, Foster S, Boyd M, et al. Family experiences of the transition to palliative care in aged residential care (ARC): A qualitative study. *International Journal of Palliative Nursing* 2017; 23: 238-247. DOI: 10.12968/ijpn.2017.23.5.238.
21. Gadoud A, Kane E, Macleod U, et al. Palliative care among heart failure patients in primary care: A comparison to cancer patients using English family practice data. *PLoS One* 2014; 9: e113188. DOI: 10.1371/journal.pone.0113188.
22. Giovannetti AM, Borreani C, Bianchi E, et al. Participant perspectives of a home-based palliative approach for people with severe multiple sclerosis: A qualitative study. *PLoS One* 2018; 13: e0200532. DOI: 10.1371/journal.pone.0200532.
23. Gomez-Batiste X, Blay C, Broggi MA, et al. Ethical challenges of early identification of advanced chronic patients in need of palliative care: The Catalan experience. *Journal of Palliative Care* 2018; 33: 247-251. DOI: 10.1177/0825859718788933.
24. Gott M, Frey R, Raphael D, et al. Palliative care need and management in the acute hospital setting: A census of one New Zealand hospital. *Bmc Palliat Care* 2013; 12: 15. DOI: 10.1186/1472-684x-12-15.
25. Gott M, Gardiner C, Ryan T, et al. Prevalence and predictors of transition to a palliative care approach among hospital inpatients in England. *Journal of Palliative Care* 2013; 29: 147-153. DOI: 10.1177/082585971302900303.
26. Gott M, Ingleton C, Gardiner C, et al. Transitions to palliative care for older people in acute hospitals: a mixed-methods study. *Health Services and Delivery Research*. Southampton (UK), 2013.
27. Grant M and Hanson J. Nursing Contributions to the Development of Palliative Care Programs. *Journal of Hospice & Palliative Nursing* 2010; 12: 319-325. DOI: 10.1097/NJH.0b013e3181eb715c.
28. Grant M, Hanson J, Mullan P, et al. Disseminating end-of-life education to cancer centers: Overview of program and of evaluation. *Journal of Cancer Education* 2007; 22: 140-148. DOI: 10.1007/BF03174326.
29. Grbich C, Parish K, Glaetzer K, et al. Communication and decision making for patients with end stage diseases in an acute care setting. *Contemporary nurse: A journal for the Australian nursing profession* 2006; 23: 21-37. DOI: 10.5172/conu.2006.23.1.21.
30. Griffiths J, Ewing G, Rogers M, et al. Supporting cancer patients with palliative care needs: District nurses' role perceptions. *Cancer Nursing* 2007; 30: 156-162. DOI: 10.1097/01.NCC.0000265013.63547.4a.
31. Grossman S. Educating RNs regarding palliative care in long-term care generates positive outcomes for patients with end-stage chronic illness. *Journal of Hospice & Palliative Nursing* 2007; 9: 323-328.
32. Hahn JE and Cadogan MP. Development and evaluation of a staff training program on palliative care for persons with intellectual and developmental disabilities. *Journal of Policy and Practice in Intellectual Disabilities* 2011; 8: 42-52. DOI: 10.1111/j.1741-1130.2011.00288.x.
33. Hall P, Hupe D and Scott J. Palliative care education for community-based family physicians: The development of a program, the evaluation, and its consequences. *Journal of Palliative Care* 1998; 14: 69-74. 1998/10/15.
34. Hall S, Goddard C, Stewart F, et al. Implementing a quality improvement programme in palliative care in care homes: A qualitative study. *BMC Geriatrics* 2011; 11: 31. DOI: 10.1186/1471-2318-11-31.
35. Hamano J, Oishi A and Kizawa Y. Identified palliative care approach needs with SPICT in family practice: A preliminary observational study. *Journal of Palliative Medicine* 2018; 21: 992-998. DOI: 10.1089/jpm.2017.0491.
36. Hendriks SA, Smalbrugge M, Deliens L, et al. End‐of‐life treatment decisions in nursing home residents dying with dementia in the Netherlands. *International Journal of Geriatric Psychiatry* 2017; 32: e43-e49. DOI: 10.1002/gps.4650.
37. Heyland Daren K, Dodek P, Rocker G, et al. What matters most in end-of-life care: Perceptions of seriously ill patients and their family members. *Canadian Medical Association Journal* 2006; 174: 627-633.
38. Hughes Philippa M, Bath Peter A, Ahmed N, et al. What progress has been made towards implementing national guidance on end of life care? A national survey of UK general practices. *Palliative Medicine* 2010; 24: 68-78.
39. Kalluri M, Claveria F, Ainsley E, et al. Beyond idiopathic pulmonary fibrosis diagnosis: Multidisciplinary care with an early integrated palliative approach Is associated with a decrease in acute care utilization and hospital deaths. *Journal of Pain and Symptom Management* 2018; 55: 420-426. DOI: 10.1016/j.jpainsymman.2017.10.016.
40. Karacsony S, Chang E, Johnson A, et al. Assessing nursing assistants' competency in palliative care: An evaluation tool. *Nurse Educ Pract* 2018; 33: 70-76. 2018/09/25. DOI: 10.1016/j.nepr.2018.09.001.
41. King N, Martin N, Bell D, et al. Now nobody falls through the net - practitioners perpectives on the gsf for community palliative care. *Palliative Medicine* 2005; 19: 619-627.
42. Krumm N, Larkin P, Connolly M, et al. Improving dementia care in nursing homes: Experiences with a palliative care symptom-assessment tool (MIDOS). *International Journal of Palliative Nursing* 2014; 20: 187-192. DOI: 10.12968/ijpn.2014.20.4.187.
43. Lau F, Downing M, Tayler C, et al. Toward a population-based approach to end-of-life care surveillance in Canada: Initial efforts and lessons. *Journal of Palliative Care* 2013; 29: 13-21.
44. Madar H, Gilad G, Elenhoren E, et al. Dialysis nurses for palliative care. *Journal of Renal Care* 2007; 33: 35-38. DOI: 10.1111/j.1755-6686.2007.tb00035.x.
45. Mahler A. The clinical nurse specialist role in developing a geropalliative model of care. *Clinical Nurse Specialist: The Journal for Advanced Nursing Practice* 2010; 24: 18-23. DOI: 10.1097/NUR.0b013e3181c4abba.
46. Mahmood-Yousuf K, Munday D, King N, et al. Interprofessional relationships and communication in primary palliative care: Impact of the Gold Standards Framework. *British Journal of Advanced Practice* 2008; 58: 256-263. DOI: 10.3399/bjgp08X279760.
47. Main J, Whittle C, Treml J, et al. The development of an Integrated Care Pathway for all patients with advanced life-limiting illness--The Supportive Care Pathway. *Journal of Nursing Management* 2006; 14: 521-528.
48. Mason B, Boyd K, Murray SA, et al. Developing a computerised search to help UK General Practices identify more patients for palliative care planning: A feasibility study. *BMC Family Practice* 2015; 16: 1-6. DOI: 10.1186/s12875-015-0312-z.
49. Matuz T, Birbaumer N, Hautzinger M, et al. Coping with amyotrophic lateral sclerosis: An integrative view. *Journal of Neurology, Neurosurgery & Psychiatry* 2010; 81: 893-898. DOI: 10.1136/jnnp.2009.201285.
50. McConigley R, Aoun S, Kristjanson L, et al. Implementation and evaluation of an education program to guide palliative care for people with motor neurone disease. *Palliative Medicine* 2012; 26: 994-1000. DOI: 10.1177/0269216311426918.
51. McVey P, McKenzie H and White K. A community‐of‐care: The integration of a palliative approach within residential aged care facilities in Australia. *Health & Social Care in the Community* 2014; 22: 197-209. DOI: 10.1111/hsc.12077.
52. Munday D, Mahmood K, Dale J, et al. Facilitating good process in primary palliative care: Does the Gold Standards Framework enable quality performance? *Family Practice* 2007; 24: 486-494.
53. Nakanishi M, Endo K, Hirooka K, et al. Dementia behaviour management programme at home: Impact of a palliative care approach on care managers and professional caregivers of home care services. *Aging Ment Health* 2017; 22: 1-6. DOI: 10.1080/13607863.2017.1332160.
54. Noble B, Hughes P, Ingleton C, et al. Impact of the Powys Macmillan GP clinical facilitator project: Views of health-care professionals. *International Journal of Palliative Nursing* 2003; 9: 528-533. DOI: 10.12968/ijpn.2003.9.12.11988.
55. O'Shea E, Timmons S, Kennelly S, et al. Symptom assessment for a palliative care approach in people with dementia admitted to acute hospitals: Results from a national audit. *Journal of Geriatric Psychiatry and Neurology* 2015; 28: 255-259. DOI: 10.1177/0891988715588835.
56. Pace A, Villani V, Di Pasquale A, et al. Home care for brain tumor patients. *Neurooncol Pract* 2014; 1: 8-12. DOI: 10.1093/nop/npt003.
57. Parker D, Clifton K, Tuckett A, et al. Palliative care case conferences in long-term care: Views of family members. *International Journal of Older People Nursing* 2016; 11: 140-148. DOI: 10.1111/opn.12105.
58. Parker D, Grbich C, Brown M, et al. A palliative approach or specialist palliative care? What happens in aged care facilities for residents with a noncancer diagnosis? *J Palliat Care* 2005; 21: 80-87.
59. Penders YWH, Albers G, Deliens L, et al. End-of-life care for people dying with dementia in general practice in Belgium, Italy and Spain: A cross-sectional, retrospective study. *Geriatrics and Gerontology International* 2017; 17: 1667-1676. DOI: 10.1111/ggi.12948.
60. Pesut B, Hooper B, Jacobsen M, et al. Nurse-led navigation to provide early palliative care in rural areas: a pilot study. *Bmc Palliat Care* 2017; 16: 37. DOI: 10.1186/s12904-017-0211-2.
61. Pesut B, Hooper B, Robinson C, et al. Feasibility of a rural palliative supportive service. *Rural & Remote Health* 2015; 15: 1-16. DOI: 3116 [pii].
62. Pesut B, McLeod B, Hole R, et al. Rural nursing and quality end-of-life care: Palliative care ... palliative approach ... or somewhere in-between? *Advances in Nursing Science* 2012; 35: 288-304. DOI: 10.1097/ANS.0b013e31826b8687.
63. Pesut B, Potter G, Stajduhar K, et al. Palliative approach education for rural nurses and health-care workers: a mixed-method study. *International Journal of Palliative Nursing* 2015; 21: 142-151. DOI: 10.12968/ijpn.2015.21.3.142.
64. Petrova M, Dale J, Munday D, et al. The role and impact of facilitators in primary care: Findings from the implementation of the Gold Standards Framework for palliative care. *Family Practice* 2009; 27: 38-47. 2009/10/31. DOI: 10.1093/fampra/cmp066.
65. Phair G, Agus A, Normand C, et al. Healthcare use, costs and quality of life in patients with end-stage kidney disease receiving conservative management: Results from a multi-centre observational study (PACKS). *Palliative Medicine* 2018; 32: 1401-1409. DOI: 10.1177/0269216318775247.
66. Phillips JL, Davidson PM, Jackson D, et al. Multi-faceted palliative care intervention: aged care nurses’ and care assistants’ perceptions and experiences. *Journal of Advanced Nursing* 2008; 62: 216-227. DOI: 10.1111/j.1365-2648.2008.04600.x.
67. Pooler C, Richman-Eisenstat J and Kalluri M. Early integrated palliative approach for idiopathic pulmonary fibrosis: A narrative study of bereaved caregivers' experiences. *Palliative Medicine* 2018; 32: 1455-1464. DOI: 10.1177/0269216318789025.
68. Potter G, Pesut B, Hooper BP, et al. Team-based education in a palliative approach for rural nurses and unlicensed care providers. *Journal of Continuing Education in Nursing* 2015; 46: 279-289. DOI: 10.3928/00220124-20150518-04.
69. Potter JM, Fernando R and Humpel N. Development and evaluation of the REACH (Recognise End of Life and Care Holistically) out in dementia toolkit. *Australasian Journal on Ageing* 2013; 32: 241-246. DOI: 10.1111/ajag.12062.
70. Ranse K, Yates P and Coyer F. Modelling end-of-life care practices: Factors associated with critical care nurse engagement in care provision. *Intensive and Critical Care Nursing* 2016; 33: 48-55. DOI: 10.1016/j.iccn.2015.11.003.
71. Rice J, Hunter L, Hsu AT, et al. Using the "Surprise Question" in nursing homes: A prospective mixed-methods study. *Journal of Palliative Care* 2018; 33: 9-18. DOI: 10.1177/0825859717745728.
72. Richards N, Ingleton C, Gardiner C, et al. Awareness contexts revisited: Indeterminacy in initiating discussions at the end-of-life. *Journal of Advanced Nursing* 2013; 69: 2654-2664. DOI: 10.1111/jan.12151.
73. Rogers MS, Barclay SIG and Todd CJ. Developing the Cambridge palliative audit schedule (CAMPAS): A palliative care audit for primary health care teams. *British Journal of General Practice* 1998; 48: 1224-1227.
74. Shahid S, Ekberg S, Holloway M, et al. Experiential learning to increase palliative care competence among the Indigenous workforce: An Australian experience. *BMJ Support Palliat Care* 2018; 9: 158-163. DOI: 10.1136/bmjspcare-2016-001296.
75. Smith D and Brown S. Integrating a palliative care approach into nursing care homes for older people. *International Journal of Palliative Nursing* 2017; 23: 511-515. DOI: 0.12968/ijpn.2017.23.10.511.
76. Solari A, Giordano A, Grasso MG, et al. Home-based palliative approach for people with severe multiple sclerosis and their carers: Study protocol for a randomized controlled trial. *Trials* 2015; 16: 184-184. DOI: 10.1186/s13063-015-0695-0.
77. Solari A, Giordano A, Patti F, et al. Randomized controlled trial of a home-based palliative approach for people with severe multiple sclerosis. *Multiple Sclerosis Journal* 2018; 24: 663-674. DOI: 10.1177/1352458517704078.
78. St John K and Koffman J. Introducing Namaste Care to the hospital environment: A pilot study. *Ann Palliat Med* 2017; 6: 354-364. DOI: 10.21037/apm.2017.06.27.
79. Stirling C, McInerney F, Andrews S, et al. A tool to aid talking about dementia and dying - Development and evaluation. *Collegian* 2014; 21: 337-343. DOI: 10.1016/j.colegn.2013.08.002.
80. Temel JS, Greer JA, Muzikansky A, et al. Early palliative care for patients with metastatic nonsmall-cell lung cancer. *New England Journal of Medicine* 2010; 363: 733-742.
81. Thomas K and Noble B. Improving the delivery of palliative care in general practice - an evaluation of the first phase of the GSF. *Palliative Medicine* 2007; 21: 49-53.
82. Thompson GN, McClement SE and Daeninck PJ. "Changing lanes": Facilitating the transition from curative to palliative care. *Journal of Palliative Care* 2006; 22: 91-98. DOI: 10.1177/082585970602200205.
83. Thoonsen B, Groot M, Engels Y, et al. Early identification of and proactive palliative care for patients in general practice, incentive and methods of a randomized controlled trial. *BMC Fam Pract* 2011; 12: 123. DOI: 10.1186/1471-2296-12-123.
84. Toye C, Jiwa M, Holloway K, et al. Can a community of practice enhance a palliative approach for people drawing close to death with dementia? *International Journal of Palliative Nursing* 2015; 21: 548-556. DOI: 10.12968/ijpn.2015.21.11.548.
85. Toye C, Robinson AL, Jiwa M, et al. Developing and testing a strategy to enhance a palliative approach and care continuity for people who have dementia: Study overview and protocol. *Bmc Palliat Care* 2012; 11: 4. DOI: 10.1186/1472-684x-11-4.
86. Walshe C, Caress A, Chew-Graham C, et al. Implementation and impact of the Gold Standards Framework in community palliative care: A qualitative study of three primary care trusts. *Palliative Medicine* 2008; 22: 736-743. DOI: 10.1177/0269216308094103.
87. Wilson SA, Kovach CR and Stearns SA. Hospice concepts in the care of end-stage dementia. *Geriatric nursing (New York, NY)* 1996; 17: 6-10. DOI: 10.1016/s0197-4572(96)80004-8.
88. Yalden J, McCormack B, O'Connor M, et al. Transforming end of life care using practice development: An arts-informed approach in residential aged care. *International Practice Development Journal* 2013; 3: 1-18.
